# Effects of Two Buckwheat Varieties on the Behavioral Choice of *Frankliniella intonsa* in Sunflower Field

**DOI:** 10.3390/insects17050523

**Published:** 2026-05-20

**Authors:** Hongxing Yang, Zerun Chuai, Jing Chang, Wenbing Zhang, Yanyan Li, Jian Zhang, Jun Zhao, Xiaopeng Yun, Haiping Li

**Affiliations:** 1 College of Horticulture and Plant Protection, Inner Mongolia Agricultural University, Hohhot 010019, China; 18822040880@163.com (H.Y.); 17614987559@163.com (Z.C.); changjing10220@imau.edu.cn (J.C.); zhangwenbing@imau.edu.cn (W.Z.); liyanyan@imau.edu.cn (Y.L.); 19995453114@163.com (J.Z.); zhaojun02@hotmail.com (J.Z.); 2 Plant Protection Department, Inner Mongolia Academy of Agriculture and Animal Husbandry, Hohhot 010020, China; y8x7peng@163.com

**Keywords:** buckwheat, sunflower, *Frankliniella intonsa*, mechanism, effect

## Abstract

Damage caused by *Frankliniella intonsa* to sunflower seeds results in the emergence of rusty speckling on the seedcoat, severely compromising seed quality in recent years. Although chemical control has remained the primary management strategy, its application during the flowering period—when *F. intonsa* is the most active—poses significant risks to pollinating insects and natural enemies, highlighting the urgent need for effective and environmentally sustainable control alternatives. Previous studies have shown that *F. intonsa* is attracted by buckwheat and that it could be a promising trap crop for *F. intonsa*. Thus, the attractiveness of *Fagopyrum esculentum* and *F. tataricum* to *F. intonsa* was compared, and the preference of *F. intonsa* between two buckwheat varieties was examined. Furthermore, the behavioral responses of *F. intonsa* to volatiles emitted by these plants in different developmental stages were assessed. The study results indicated that *F. intonsa* had a clear preference for *F. tataricum* over *F. esculentum*. In cage trials, the selection rates of 2nd instar nymphs and adults of *F. intonsa* for *F. tataricum* were 61.63% and 60.19% at the seedling stage, and 60.74% and 62.50% at the full-bloom stage, all significantly surpassing those of *F. esculentum*. Olfactory bioassays further confirmed that flowers of *F. tataricum* were notably more appealing to both 2nd instar nymphs and adults of *F. intonsa*, with selection rates of 64.17% and 61.67%, respectively. Twenty distinct floral volatiles of two buckwheat varieties were detected through the phytochemical analysis. Orthogonal partial least squares-discriminant analysis (OPLS-DA) identified seven key compounds that accounted for the observed behavioral differences. Both 2nd instar nymphs and adults of *F. intonsa* demonstrated a significant selection for Δ-Cadinene, with the highest selection rates of 75.00% and 76.67% recorded at a concentration of 0.1 μg/μL. Furthermore, *F. intonsa* exhibited a marked attraction to higher concentrations of Verbenone, which was unique to *F. tataricum*, and (S)-2-Methyl-1-butanol, which was unique to *F. esculentum*. Field intercropping experiments confirmed that *F. tataricum* outperformed *F. esculentum* in trapping *F. intonsa* within sunflower plots. In conclusion, the results indicated that *F. tataricum* possessed considerable potential as a trap crop for the integrated management of *F. intonsa* in sunflower cultivation systems.

## Simple Summary

*Frankliniella intonsa* is an important thrip pest. Although chemical control remains the primary management strategy, its application during the flowering period—when *F. intonsa* is the most active—poses significant risks to pollinating insects and natural enemies, highlighting the urgent need for effective and environmentally sustainable control alternatives. The attractiveness of *Fagopyrum esculentum* and *Fagopyrum tataricum* to *F. intonsa* was compared, and the preference of *F. intonsa* between two buckwheat varieties was examined. Furthermore, the behavioral responses of *F. intonsa* to volatiles emitted by these plants in different developmental stages were assessed. The study results indicated that *F. intonsa* had a clear preference for *F. tataricum* over *F. esculentum*. Field intercropping experiments confirmed that *F. tataricum* outperformed *F. esculentum* in trapping *F. intonsa* within sunflower plots. In conclusion, the results show that *F. tataricum* possesses considerable potential as a trap crop for the integrated management of *F. intonsa* in sunflower cultivation systems.

## 1. Introduction

Thrips (Thysanoptera: Thripidae) are a major global pest of many plants. Thrips may cause direct cosmetic damage to plants in addition to the more severe damage caused by their ability to transmit viruses to the crop [1,2,3]. In recent years, the damage caused by thrips has been increasing in China. In 2023, vegetable thrips were included in the List of Crop Pests of Category I of the Ministry of Agriculture and Rural Affairs of the People’s Republic of China for the first time (Announcement No. 654) [4].

Flower thrips (*Frankliniella intonsa* (Trybom, 1895) (Thysanoptera: Thripidae)) are an invasive and polyphagous pest. Flower thrips directly damage many commercial food crops by feeding on leaves, petals, fruits or pollen of green plants, thereby affecting their yield or cosmetic appearance [5]. Chemical control is currently the most commonly employed prevention and control method in China. It has become more difficult to control flower thrips effectively due to their development of resistance to chemical insecticides [6]. In recent years, *F. intonsa* have been widely found in major sunflower-producing regions, such as Inner Mongolia, Xinjiang, Gansu, among others, causing seedcoat rust and significantly reducing seed quality [7,8,9]. The commodity value of sunflower seeds with rust spots decreases, resulting in a decrease in their purchase price and an economic loss to farmers. At present, management of *F. intonsa* relies heavily on chemical control to reduce thrips’ settling and/or feeding on the sunflower [10]. However, *F. intonsa* often hide in the buds and on the back of leaves, making it difficult to achieve the desired effect with chemical control. *F. intonsa* frequently occur at high levels during sunflowers’ flowering period, and chemical control also harms pollinating insects and natural enemies [10]. Therefore, it is crucial to develop green control measures for *F. intonsa* to reduce the occurrence of sunflower seedcoat rust.

Current pest control methods are mainly based on the concept of IPM (Integrated Pest Management). Various control methods are combined to reduce the negative impacts on the ecological environment and achieve effective pest control [11]. In the IPM framework, the behavior of target pests is influenced by trap plants to reduce their harm to the main crops, resulting in pest regulation. This method can not only effectively control pests, but also reduces the use of chemical pesticides and improves the diversity of the farmland ecosystem [12]. In current agricultural practices, there are successful examples of trap plants being used to control pests. In 1860, Americans introduced intercropping with alfalfa to attract *Lygus hesperus* (Knight, 1926) (Hemiptera: Miridae) in cotton fields (at the University of California Cotton Research Station near Shafter), and this planting pattern continues to be used in America today [13]. In modern agriculture, the diversification of crop planting plays an increasingly important role, and improving the diversity of farmland landscapes is a key strategy for improving pest management [14,15].

Buckwheat is a common plant for attracting pests and serves as a nectar source. Cultivated varieties mainly include sweet buckwheat (*Fagopyrum esculentum*) (Moech, 1794) (Caryophyllales: Polygonaceae) and bitter buckwheat (*F. tataricum*) ((L.) Gaertn, 1791) [16,17]. Buckwheat plants are relatively short, with clusters of inflorescences that last for a long period, accounting for about two-thirds of the plant’s life cycle. Buckwheat has strong resistance and a short growth cycle, making it widely cultivated in Inner Mongolia, where it is one of the common coarse grains. Buckwheat is also an important host plant for *F. intonsa* [18,19,20]. Therefore, based on practical production experience, two common buckwheat varieties were selected as trapping plants in Inner Mongolia in this study. How attractive these two buckwheat varieties are to *F. intonsa*, the primary volatile components of the buckwheat flowers (*F. esculentum* and *F. tataricum*), and *F. intonsa*’s attraction levels to these components in sunflower fields were systematically compared. This study aims to provide a theoretical basis for the green control of *F. intonsa* in sunflower fields, promoting the healthy development of the sunflower industry.

## 2. Materials and Methods

### 2.1. Insects

A natural colony of *F. intonsa* was collected from sunflower fields in Wuyuan County, Bayannoer City, Inner Mongolia, China, and reared in climate-controlled rooms on cowpea pods until the F_8_ generation was obtained. The rearing conditions maintained a temperature of 25 ± 1 °C, a relative humidity of 50–60% RH, a photoperiod of 16L:8D, and an intensity of 18,000 lx.

### 2.2. Crops

Confection sunflower (variety SH361), *F. esculentum* (variety Moench 0208, the flower is red), and *F. tataricum* (variety Jinqiaomai 6, the flower is white) were used.

### 2.3. Experimental Fields

This study was conducted in two experimental fields in Ulat Banner (108°52′ E, 40°93′ N) (2023) and Wuyuan County, Bayannoer City (108°6′58.59′′ E, 41°3′51.46′′ N) (2024), Inner Mongolia, China.

### 2.4. Selection of Two Types of Buckwheat by F. intonsa in Cages

The cage assay followed Zhou’s methodology [21]. Experiments were conducted in insect-rearing cages measuring 50 × 50 × 50 cm. Each pot of uniformly growing *F. esculentum* and *F. tataricum* was carefully selected, with the exterior of the pots wrapped in polyethylene film to minimize external disturbance. The pots were positioned 5 cm from the diagonally opposite corners of the cage floor. A total of 50 individuals of *F. intonsa* were introduced into a glass Petri dish (5 cm diameter) placed at the center of the cage. This arrangement provided free access for *F. intonsa* to both buckwheat species. The 2nd instar nymphs and newly unmated adults (individually reared the 2nd instar nymphs) were starved for 4 h in advance [22,23]. After 24 h, the number of *F. intonsa* settled on each plant was recorded, and the experiment was replicated six times (twelve pots *F. esculentum* and twelve pots *F. tataricum*).

### 2.5. Olfactory Behavior of Two Stages of F. intonsa on Different Parts of Buckwheat

The olfactory response of *F. intonsa* was evaluated using a Y-tube olfactometer, following the procedure described by Chen et al. [24]. The glass Y-tube olfactometer consisted of a 115-mm-long base tube and two 190-mm-long arms separated at an angle of 60°. The internal diameter of the base tube and arms was 8 mm. Air reentered the sample glass flask and into one arm of the olfactometer at a rate of 200 mL/min controlled by a float-type flowmeter. All experiments occurred in a darkroom maintained at 25 ± 1 °C and 50 ± 5% relative humidity from 12:00 to 15:00 every day [25]. To eliminate visual interference, a 100 lx LED light source was positioned above the olfactometer. Prior to testing, uniformly grown buckwheat plants at the seedling stage were selected, and 5 g of healthy leaves were harvested. Additionally, 5 g of inflorescences was collected from plants at full bloom. Adult *F. intonsa*, starved for 4 h, were used in the assays. The choice of *F. intonsa* for one of the two odour sources was recorded when it crossed a half-length of either arm within 2 min. If *F. intonsa* remained in the base tube, it was recorded as no response. *F. intonsa* that did not make a choice within this period were replaced. Each experimental group consisted of 20 *F. intonsa* displaying a definitive behavioral choice, with 6 replicates (total n = 120). The positions of the Y-tube arms were switched after every 5 *F. intonsa*, the Y-tube was rotated 180° along the entry tube and the experiment was continued to avoid position effects. After 10 *F. intonsa* made their choices, the equipment was cleaned with 95% ethanol. The selection rate was calculated according to the following formula:

Selection rate (%) = (Number of *F. intonsa* exhibiting a choice/Total number of *F. intonsa* assayed) × 100% (1)

### 2.6. Preference of F. intonsa Between Two Colors of Buckwheat Flowers

Flowers of red-flowered (*F. esculentum*) and white-flowered (*F. tataricum*) buckwheat underwent digital scanning (Epson Perfection V10; Epson (China) Co., Ltd., Beijing, China), and the resulting images were stored for subsequent analysis. Utilizing the RGB color model as described by Byers [26], floral colors were simulated in Photoshop 2021. The RGB values for red-flowered buckwheat were (255, 178, 248), while those for white-flowered buckwheat were (254, 255, 245). Corresponding colored cards were printed based on these values, and both sides of each card received a coating of eco-friendly hot-melt adhesive. The colored cards were then trimmed into rectangular strips measuring 5 cm × 3 cm. Two cards of different colors were attached diagonally to the inner wall of an 11 cm diameter Petri dish. The 1.5 mL microcentrifuge tube containing 40 individuals of *F. intonsa* (2nd instar nymphs or adults starved for 4 h) was placed at the center of the dish, ensuring equal access to both colored cards. Each dish was sealed with polyethylene film, which was perforated 50–60 times with a 0.02 cm diameter syringe needle to maintain airflow. All assays were conducted in a growth chamber under the conditions described in Section 2.1. After 1 h, the number of *F. intonsa* attracted to each colored card was recorded. Each treatment was replicated 6 times.

### 2.7. Volatile Collection and Analysis

#### 2.7.1. Two Types of Buckwheat Volatile Collection

Based upon established methodologies, floral volatile compounds from two species of buckwheat (*F. esculentum* and *F. tataricum*) were collected using Headspace Solid-Phase Microextraction (HS-SPME) [27], as reported by Qu et al. [20]. Each variety of buckwheat involved the extraction of 3 g of fully opened flowers. The materials were placed separately into 20 mL bottles to collect headspace volatiles, with empty vials serving as negative controls. Volatiles were collected on SPME fibers coated with 100 μm of polydimethylsiloxane at 25 ± 1 °C for 40 min. The collection process for the flowers of both buckwheat varieties and the control group was repeated 3 times under identical parameters to eliminate interfering impurities from the air.

#### 2.7.2. Two Types of Buckwheat Chemical Analysis (GC-MS)

Volatile compounds collected from two buckwheat flower varieties via Headspace Solid-Phase Microextraction (HS-SPME) were analyzed using Gas Chromatography–Mass Spectrometry (GC-MS). The analysis utilized a Thermo Scientific system equipped with an HP-5 chromatographic column (Agilent (Research and Development Center, Beijing, China); 30 m × 0.32 mm ID, 0.25 μm film thickness). High-purity helium gas at a flow rate of 1.0 mL/min was used, and the injector temperature was set to 250 °C. The heating procedure commenced at an initial temperature of 50 °C and was held for 4 min; this was followed by an increase to 150 °C at a rate of 6 °C/min and maintained for 1 min. Subsequently, the temperature rose to 250 °C at a rate of 7 °C/min and was held for 3 min. Mass spectrometric detection occurred in electron ionization (EI) mode at 70 eV, with a scan range of 30–350 *m*/*z*. The volatile compounds were identified by comparing their mass spectra with the NIST mass spectra library (NIST/EPA/NIH, 2014), and the relative contents of compounds were calculated using the area normalization method. The retention times of compounds and mass spectra were compared with those of synthetic standards. Each compound’s retention index was calculated using a C7-C30 alkane standard mix (Sigma-Aldrich, St. Louis, MO, USA). Three replicate analyses were conducted for each buckwheat flower variety. On this basis, the orthogonal Partial Least Squares Discriminant Analysis (PLS-DA) method was used to analyze the specific compounds of two kinds of buckwheat inflorescences [28]. The plots of scores of variable importance in projection (VIP) were obtained from PLS-DA [29]. The formula for calculating the VIP value is as follows:

$${\mathrm{VIP}}_{j}=\sqrt{\frac{p\sum_{a=1}^{A}(W_{\mathrm{ja}}^{2}\cdot {\mathrm{SSY}}_{a})}{\sum_{a=1}^{A}{\mathrm{SSY}}_{a}}}$$

p: Total number of predictive variables; A: Number of PLS components; W_ja_: The weight of the j-th variable in the a-th PLS component; SSY_a_: The variance explained by the a-th PLS component for the dependent variable; VIP_j_: The VIP value of the j-th variable.

#### 2.7.3. Y-Tube Olfactometer Bioassays

Based on preliminary experimental results, standard volatile compounds were prepared as test samples at three concentration gradients (0.1, 1, and 10 μg/μL) using liquid paraffin as the solvent. The Y-tube olfactometer evaluated the orientation behavior of *F. intonsa* toward these volatiles across the specified concentration gradients. Prior to each assay, 40 μL of the test solution was applied to a 2 cm diameter circular filter paper, with an equivalent volume of liquid paraffin serving as the control. The researchers replaced the filter papers every 10 min, simultaneously replenishing both the test compounds and liquid paraffin. The experimental protocol adhered to the procedure outlined in Section 2.4, with six replicates per concentration and 20 individuals of *F. intonsa* used per replicate.

### 2.8. Field Layout and Crop Planting

**Figure 1:**
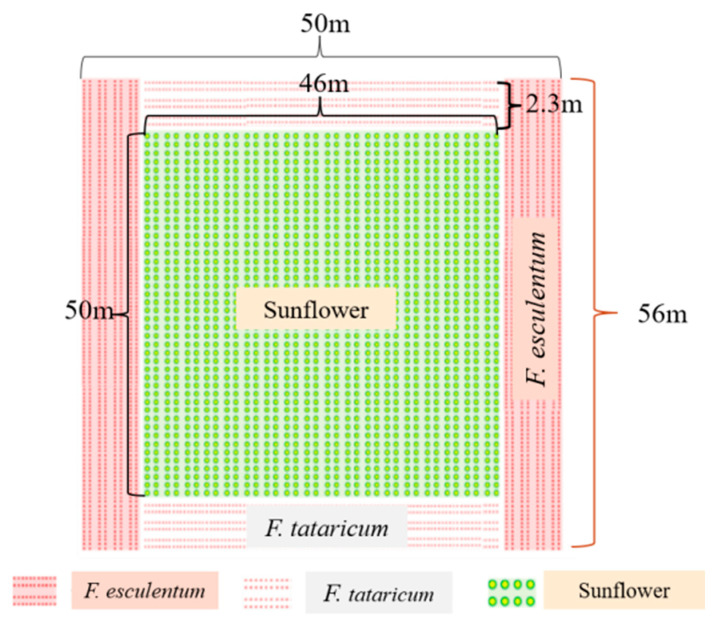
A schematic diagram of the experimental sunflower field with buckwheat border planting in Wuyuan County.

In 2023, the total area measured approximately 2800 m^2^ (50 m × 56 m), which included about 220 m^2^ of *F. esculentum*, 212 m^2^ of *F. tataricum*, and 2300 m^2^ of edible sunflower. In 2024, the total area decreased to approximately 2350 m^2^, comprising roughly 244 m^2^ of *F. esculentum*, 198 m^2^ of *F. tataricum*, and 1900 m^2^ of sunflower. There were two treatments—sunflower monoculture and sunflower-buckwheat intercropping (Figure 1)—and each treatment was replicated three times. All plots were cultivated using plastic film mulch, with an inter-film spacing of 1.1 m. Two crop rows were arranged per film strip, maintaining an inter-row spacing of 0.6 m. Sunflowers were planted with an intra-row spacing of 0.6 m (1 seed per hole), while buckwheat was established at an intra-row spacing of 0.5 m (3–5 seeds per hole). *F. tataricum* and *F. esculentum* were positioned on opposite sides of the sunflower plots. Buckwheat was sowed earlier 3 d than sunflower, in order to have the buckwheat flowering match with the sunflower R4 stage; thus, the maximum trapping effect of buckwheat should be presented at the critical stage of sunflower development.

Blue sticky traps (characteristic wavelength: 460 ± 10 nm) were deployed to monitor the population dynamics of *F. intonsa*. In the buckwheat zones, traps were mounted on bamboo poles inserted 1.4 m from the edges of the sunflower plots. They were suspended 0.2 m above the buckwheat (or sunflower) canopy, maintaining a consistent inter-trap distance of 7 m. Sunflower growth stages were systematically classified according to the criteria established by Schneiter et al. [30]. The sticky traps were replaced at five-day intervals, and the number of *F. intonsa* captured on the collected sticky traps was recorded.

The sunflowers and buckwheat utilized for behavioral testing and volatile compound collection were cultivated in the experimental area in fields that did not employ any pesticides. The experiment and data analysis occurred during a designated test period established according to Schneiter’s classification standard for the reproductive stage of sunflowers.

### 2.9. Statistical Analyses

All statistical datasets were processed and organized using Microsoft Excel 2016. Data analysis was conducted with the SPSS 26.0 software (IBM, Armonk, NY, USA). For the olfactometer behavioral assays and cage trials of *F. intonsa*, a *x*^2^ goodness-of-fit test evaluated deviations from the expected 50:50 distributions, with *x*^2^ and *p* values calculated accordingly. The trapping efficacy of two buckwheat species against *F. intonsa* and population dynamics across distinct cultivation regions was assessed using Student’s *t* test (percentage data were arcsine square root-transformed for further statistical analysis). Figures were constructed with GraphPad Prism version 10.1.2.

## 3. Results

### 3.1. Statistical Analysis of the Number of F. intonsa in the Cage

**Figure 2:**
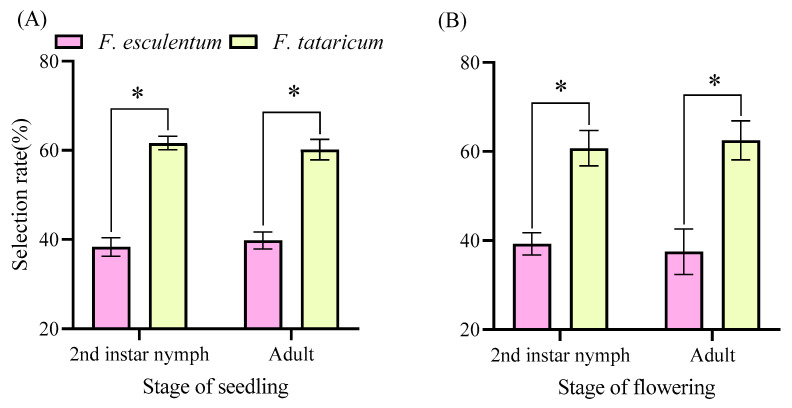
Preference of *F. intonsa* between *F. tataricum* and *F. esculentum* in cage. Note: (**A**) shows the seedling stage of buckwheat. (**B**) shows the flowering stage of buckwheat. * indicates that there is a significant difference between the processing groups (*p* < 0.05).

*F. intonsa* exhibited significantly higher selection for *F. tataricum* compared to *F. esculentum* across all growth stages in cage trials (Figure 2). At the seedling stage, both 2nd instar nymphs (61.63%, *x*^2^ = 5.760, *p* = 0.016) and adults (60.19%, *x*^2^ = 4.000, *p* = 0.046) demonstrated a significantly greater selection for *F. tataricum* (*p* < 0.05; Figure 2A). A consistent selection pattern persisted during the full-bloom stage, as *F. intonsa* continued to show significant selection toward *F. tataricum* flowers (*p* < 0.05; Figure 2B).

### 3.2. Olfactory Behavioral Responses of F. intonsa to Two Types of Buckwheat

**Figure 3:**
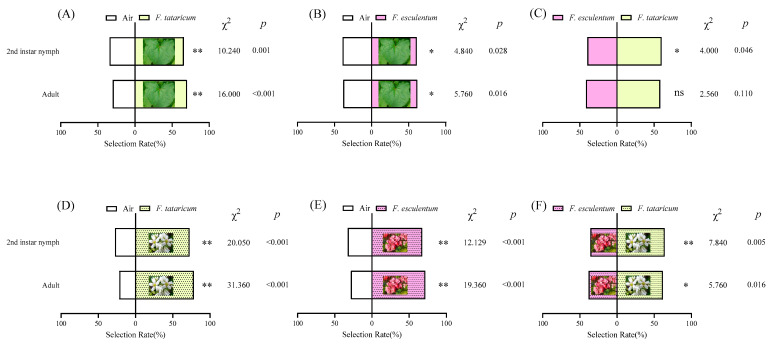
Preference of *F. intonsa* for leaves and flowers of *F. tataricum* and *F. esculentum.* Note: (**A**) represents the selection of the *F. intonsa* between the leaves of *F. tataricum* and the air. (**B**) represents the selection of the *F. intonsa* between *F. esculentum* leaves and air. (**C**) represents the selection of the *F. intonsa* between *F. tataricum* leaves and *F. esculentum* leaves. (**D**) represents the selection of the *F. intonsa* between the *F. tataricum* flower and the air. (**E**) represents the selection of the *F. intonsa* between *F. esculentum* flowers and air. (**F**) represents the selection of the *F. intonsa* between *F. tataricum* flowers and *F. esculentum* flowers. ** indicates that there is a significant difference between the processing groups (*p* < 0.01), * indicates that there is a significant difference between the processing groups (*p* < 0.05), and ns indicates no significant difference.

Significant differences emerged in the selection rates of *F. intonsa* for buckwheat leaves compared to air, as well as between the two varieties of buckwheat leaves (*p* < 0.05; Figure 3). Leaf volatiles from both buckwheat varieties exhibited significant attractant effects on *F. intonsa* across various developmental stages when compared to air (*p* < 0.05; Figure 3A,B). Specifically, both 2nd instar nymphs and adults of *F. intonsa* demonstrated markedly higher selection rates for *F. tataricum* leaves (65.83%, *x*^2^ = 10.240, *p* = 0.001, and 70.00%, *x*^2^ = 16.000, *p* < 0.001, respectively), which were significantly greater than those for air (*p* < 0.05). In dual-choice assays between the two buckwheat varieties, the 2nd instar nymphs displayed a significant preference for *F. tataricum* leaves (60.00%, *x*^2^ = 4.000, *p* = 0.046) over *F. esculentum* leaves (40.00%; *p* < 0.05). In contrast, adult *F. intonsa* showed no significant preference between the two leaf types (*x*^2^ = 2.560, *p* = 0.110, *p* > 0.05; Figure 3C).

The olfactory behavioral responses of *F. intonsa* to the two types of buckwheat flowers demonstrated significant differences (Figure 3D–F). Compared to the air control, *F. intonsa* exhibited markedly higher selection rates for both types of buckwheat flowers (*p* < 0.01; Figure 3D,E). In binary choice assays between the two buckwheat flower varieties, *F. intonsa* displayed a distinct preference for *F. tataricum* flowers over *F. esculentum* flowers (Figure 3F). Specifically, adults *F. intonsa* significantly preferred *F. tataricum* flowers (61.67%, *x*^2^ = 5.760, *p* = 0.016) to *F. esculentum* flowers (38.33%; *p* < 0.05), while 2nd instar nymphs showed an even more pronounced preference for *F. tataricum* flowers (64.17%, *x*^2^ = 7.840, *p* = 0.005) over *F. esculentum* flowers (35.83%; *p* < 0.01).

### 3.3. Choice of F. intonsa Between Two Color Palettes

*F. intonsa* exhibited statistically significant differences in the selection of color cards representing the two buckwheat flower types. A greater number of *F. intonsa* chose the white-flower-colored cards compared to the red-flower-colored cards (*p* < 0.05; Table 1). The difference in color card selection by 2nd instar nymphs proved highly significant (*p* < 0.01), while the selection demonstrated by adults was statistically significant (*p* < 0.05).

### 3.4. Buckwheat Flower Volatile Compounds

#### 3.4.1. OPLS-DA for Two Types of Volatile Compounds in Buckwheat Flower Clusters

**Figure 4:**
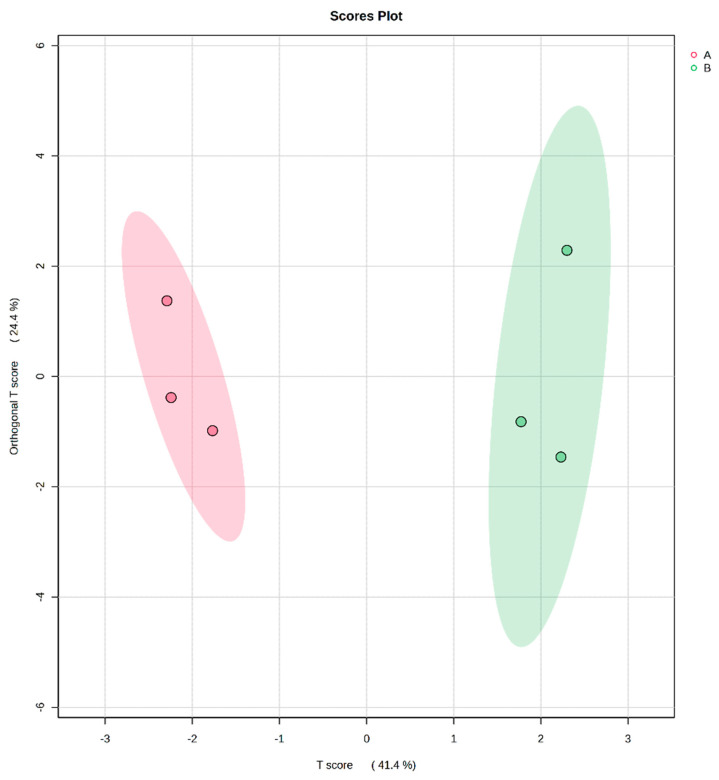
The scores plot of *F. tataricum* and *F. esculentum* volatile components based on OPLS-DA. Note: The red sample is *F. esculentum*, and the green sample is *F. tataricum*.

A total of 20 volatile compounds from the blossoms of both buckwheat varieties, respectively, were identified using GC-MS. The peak volumes of volatile compounds that met the threshold greater than 0.1% in relative content were quantified and summed, encompassing a total of 13 compounds, among which 8 were identified as co-eluting volatiles shared between the two buckwheat varieties. Orthogonal Projections to Latent Structures-Discriminant Analysis (OPLS-DA), implemented in SIMCA 14.0, was applied to evaluate the combined volatile profiles (Figure 4). The OPLS-DA results revealed a clear separation between the red-flowered and white-flowered buckwheat samples, with the predictive component (T score) accounting for 41.4% of the variation and the Orthogonal T score accounting for 24.4%. This indicated discernible differences in both the relative abundance and composition of volatiles between the two buckwheat varieties. The model demonstrated a high goodness-of-fit, with R^2^Y = 0.968 and Q^2^ = 0.885. The condition R^2^Y > Q^2^ confirmed the model’s robustness without over-fitting, thereby supporting its reliability for subsequent screening of key discriminatory volatiles. R^2^Y reflects the model’s ability to fit the dependent variable Y; Q^2^ measures the predictive capability of the model. R^2^Y and Q^2^ values greater than 0.5 indicate that the fit results are acceptable. The values closer to 1 indicates a stronger explanatory power and predictive performance of the model.

**Figure 5:**
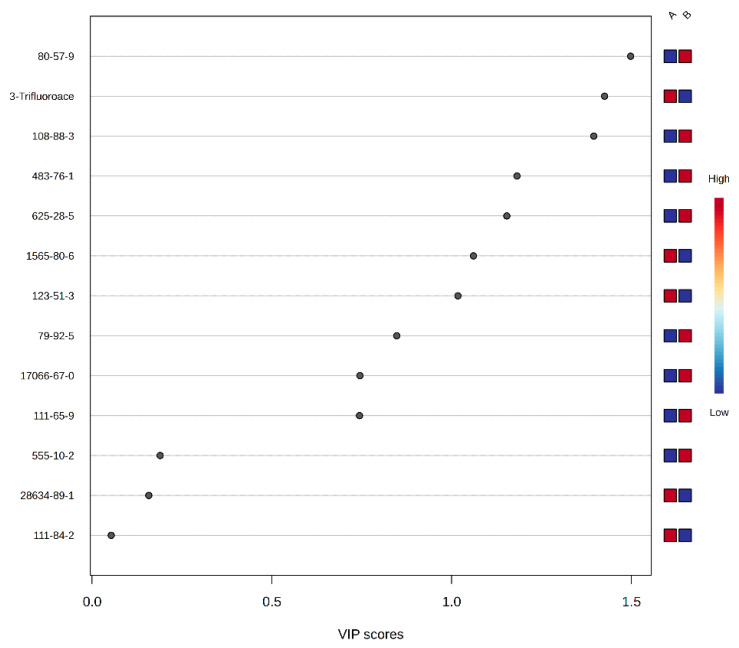
Volatile components of variable importance in the projection scores in the OPLS-DA model. Note: VIP values greater than 1 were considered key variables, and VIP values lower than 0.5 were considered non-key variables.

The Variable Importance in Projection (VIP) analysis is shown in Figure 5. The analysis indicated that 10 volatile compounds had VIP scores exceeding 0.5, with the exceptions of n-Nonane, β-Thujene, and β-Phellandrene. Seven compounds—Verbenone, 3-Trifluoroethoxyoctadecane, Toluene, Δ-Cadinene, Isovaleronitrile, (S)-2-Methyl-1-butanol, and Isoamyl alcohol—demonstrated VIP values greater than 1.0, signifying their importance as key discriminatory markers in the floral volatiles of the two buckwheat varieties.

#### 3.4.2. Responses of *F. intonsa* to the Synthetic Standards of Major Volatile Compounds

An integrated analysis that combined Orthogonal Projections to Latent Structures-Discriminant Analysis (OPLS-DA) and Variable Importance in Projection (VIP) examined seven volatile compounds in the flowers of two types of buckwheat. This analysis identified five key volatile compounds, Δ-Cadinene, Verbenone, (S)-2-Methyl-1-butanol, Isopentyl alcohol, and Isovaleronitrile, used in behavioral orientation assays with 2nd instar nymphs and adults of *F. intonsa*.

**Figure 6:**
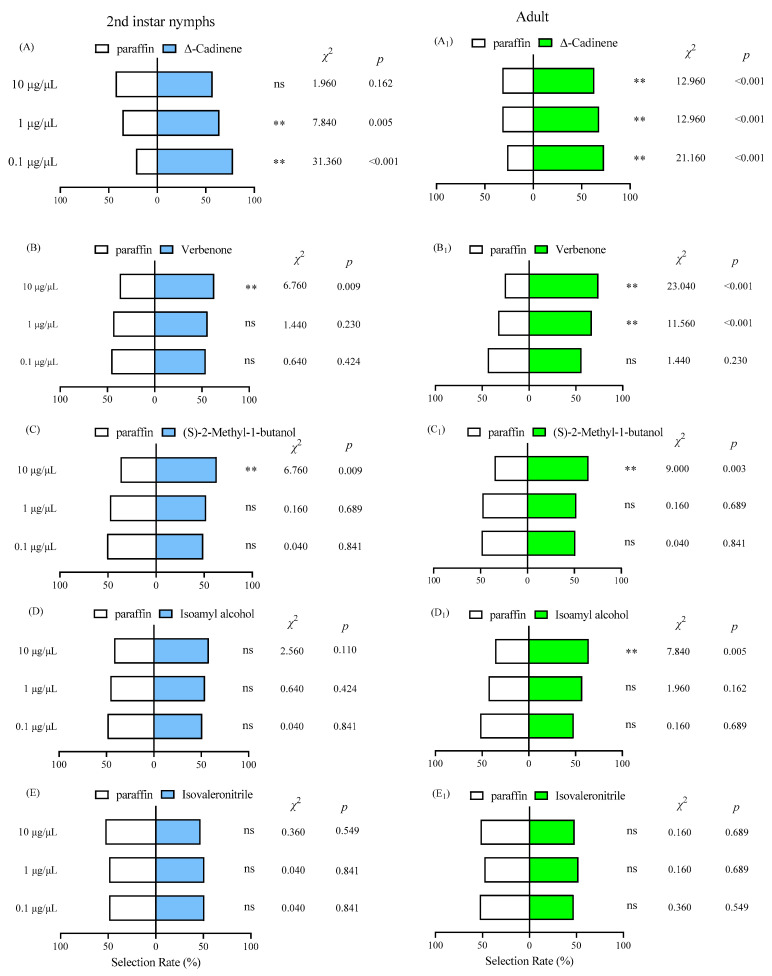
Responses of adults and nymphs of *F. intonsa* to important volatile components of buckwheat. Note: (**A**) represents the selection of 2nd instar nymphs between air and Δ-Cadinene. (**A_1_**) represents the selection of adult between air and Δ-Cadinene. (**B**) represents the selection of 2nd instar nymphs between air and Verbenone. (**B_1_**) represents the selection of adult between air and Verbenone. (**C**) represents the selection of 2nd instar nymphs between air and (S)-2-Methyl-1-butanol. (**C_1_**) represents the selection of adult between air and (S)-2-Methyl-1-butanol. (**D**) represents the selection of 2nd instar nymphs between air and Isopentyl alcohol. (**D_1_**) represents the selection of adult between air and Isopentyl alcohol. (**E**) represents the selection of 2nd instar nymphs between air and Isovaleronitrile. (**E_1_**) represents the selection of adult between air and Isovaleronitrile. ** indicates that there is a significant difference between treatment and check (*p* < 0.01), and ns indicates no significant difference, the same as below.

Both adults and 2nd instar nymphs of *F. intonsa* exhibited significant differences in their behavioral selection toward the five volatile compounds, with concentration-dependent effects observed for these compounds (Figure 6). Among the five volatiles tested, Isovaleronitrile elicited no significant behavioral response in 2nd instar nymphs of *F. intonsa* across the evaluated concentration range (0.1 µg/µL: *x*^2^ = 0.040, *p* = 0.841; 1 µg/µL: *x*^2^ = 0.040, *p* = 0.841; 10 µg/µL: *x*^2^ = 0.360, *p* = 0.549; *p* > 0.05; Figure 6E). In contrast, Isopentyl alcohol at 10 µg/µL significantly attracted adult *F. intonsa* (*x*^2^ = 7.840, *p* = 0.005; *p* < 0.01; Figure 6D_1_), although it did not affect 2nd instar nymphs. The remaining three compounds significantly attracted *F. intonsa* at specific concentrations. Notably, Δ-Cadinene exhibited the most pronounced attractant activity (Figure 6A); among the three concentrations tested, both adults (0.1 µg/µL: *x*^2^ = 21.160, *p* < 0.001; 1 µg/µL: *x*^2^ = 12.960, *p* < 0.001; 10 µg/µL: *x*^2^ = 12.960, *p* < 0.001) and 2nd instar nymphs (0.1 µg/µL: *x*^2^ = 31.360, *p* < 0.001; 1 µg/µL: *x*^2^ = 7.840, *p* = 0.005) were highly significantly attracted (*p* < 0.01) at all concentrations except 10 µg/µL (*x*^2^ = 1.960, *p* = 0.162), which had no significant effect on 2nd instar nymphs. Both Verbenone (Figure 6B, *x*^2^ = 6.760, *p* = 0.009 and Figure 6B_1_, *x*^2^ = 23.040, *p* < 0.001) and (S)-2-Methyl-1-butanol (Figure 6C, *x*^2^ = 0.04, *p* = 0.841 and Figure 6C_1_, *x*^2^ = 9.000, *p* = 0.003) had a highly significant attractant effect (*p* < 0.01) on adults and 2nd instar nymphs at 10 μg/μL; Verbenone also exerted equally strong effects on adults at 1 μg/μL (*x*^2^ = 11.560, *p* < 0.001).

### 3.5. Effect of Two Types of Buckwheat on Population Dynamics of F. intonsa

**Figure 7:**
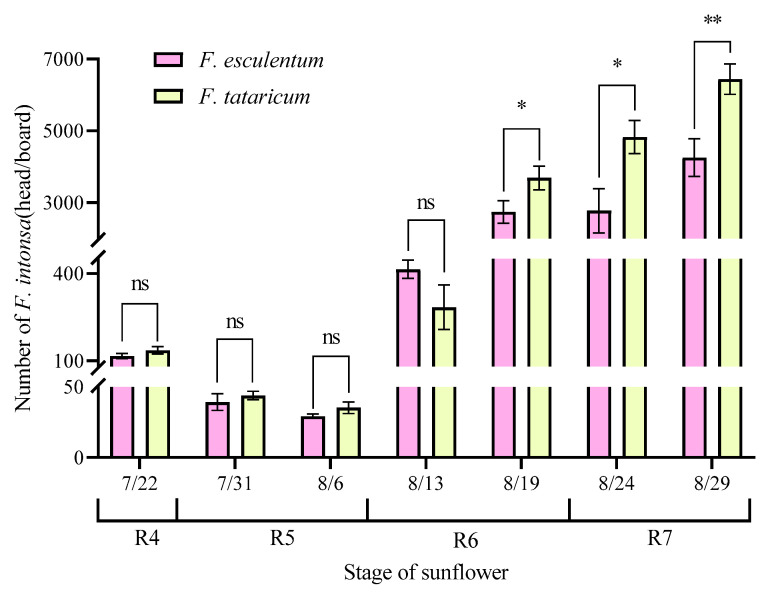
The number of *F. intonsa* attracted by *F. tataricum* and *F. esculentum.* Note: * indicates that there is a significant difference between treatment and check (*p* < 0.05), ** indicates that there is a significant difference between treatment and check (*p* < 0.01), and ns indicates no significant difference, the same as below.

Field trials revealed stage-dependent differences in the trapping efficacy of two buckwheat varieties against *F. intonsa* (Figure 7). During the sunflower R4 to R6 growth stages (22 July to 13 August), thrip population densities remained relatively low on both *F. tataricum* and *F. esculentum*, with no significant differences in captures on blue sticky traps between the two varieties (*p* > 0.05). As the sunflower progressed into the late R6 and R7 stages, thrip populations increased substantially. Notably, the number of *F. intonsa* captured in *F. tataricum* plots (3686.33–6437.00 heads per trap) significantly exceeded that in *F. esculentum* plots (8/19: *t* = 3.590, *p* = 0.023; 8/24: *t* = 4.604, *p* = 0.010; 9/24: *t* = 5.621, *p* = 0.005; *p* < 0.05).

### 3.6. Effect of Sunflower/Buckwheat Intercropping on Population Dynamics of F. intonsa

**Figure 8:**
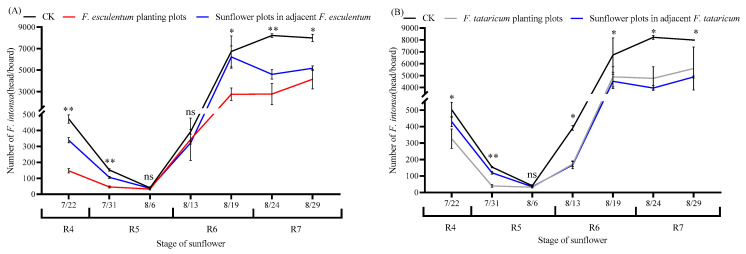
Dynamics of occurrence of *F. intonsa* in different planting areas. Note: (**A**) represents *F. esculentum* and the adjacent sunflower planting area near *F. esculentum*, (**B**) represents *F. tataricum* and the adjacent sunflower planting area near *F. tataricum*. * indicates that there is a significant difference between treatment and check (*p* < 0.05), ** indicates that there is a significant difference between treatment and check (*p* < 0.01), and ns indicates no significant difference, the same as below.

Perimeter planting with two buckwheat varieties significantly reduced the population densities of *F. intonsa* in adjacent sunflower plots (Figure 8). Throughout all observed sunflower flowering stages, the number of *F. intonsa* captured in control plots remained consistently higher than that in treatment plots. Furthermore, the density of *F. intonsa* captured varied according to the species of buckwheat planted adjacent to sunflowers in the treatment groups, indicating a differential effect between *F. tataricum* and *F. esculentum*.

In the buckwheat perimeter-planting treatments, sunflower plots adjacent to *F. esculentum* generally exhibited higher thrip population densities than the corresponding buckwheat plots across most growth stages, except during the late R5 to early R6 period (6–13 August). Notably, during the late R6 stage (19 August), the density of *F. intonsa* in sunflower plots adjacent to *F. esculentum* (6221.00 heads per trap) significantly exceeded that in the buckwheat plots (2753.00 heads per trap; *t* = 5.056, *p* = 0.013, *p* < 0.01; Figure 8A). In plots bordered by *F. tataricum*, the number of thrips in sunflower areas was significantly higher than that in the adjacent buckwheat during the R4 (*F. tataricum* plots: *t* = 4.116, *p* = 0.015) to R5 (*F. tataricum* plots: *t* = 21.756, *p* < 0.001) stages, except on August 6 (*p* < 0.05; Figure 8B). However, beginning 6 August, thrips captured in *F. tataricum* plots consistently surpassed those in adjacent sunflower plots. The peak population density occurred earlier in the treatment groups, reaching its maximum during the late R6 stage (19 August), whereas control plots peaked later, in the early R7 stage (24 August). As the ray and disk florets of sunflowers progressively senesced, thrip populations increased across all planting arrangements during the late R6 (19 August) and R7 stages. Furthermore, population densities in sunflower plots adjacent to *F. tataricum* (3960.33–4885.33 heads per trap) remained consistently lower than those near *F. esculentum* (4608.00–6221.00 heads per trap).

## 4. Discussion

*F. intonsa* exhibits a broad host range as a polyphagous herbivorous insect, yet it demonstrates distinct selection among potential host plants [31]. Buckwheat (*F. esculentum*) served as a significant host, for which *F. intonsa* showed a notable preference [19,20]. Consistent with its pronounced anthophilous behavior, the experimental results indicated that buckwheat at the flowering stage attracted *F. intonsa* more effectively than at the seedling stage. Furthermore, significant differences emerged between two buckwheat varieties with differing floral pigmentation: inflorescences of *F. tataricum* flowers attracted 2nd instar nymphs and adults of *F. intonsa* significantly more than those of *F. esculentum* flowers. This divergence likely stemmed from differences in floral coloration, suggesting varied sensitivity and behavioral responses of *F. intonsa* to specific light wavelengths. Such variation in sensitivity and phototaxis to different colors and wavelengths is commonly observed among insects [32,33]. Previous studies have reported that thrips species exhibited distinct color preferences toward host plants [34]. Lopez-Reyes et al. explain that thrips are highly attracted to green (532 nm) with spectral curves [35]. In the present study, RGB simulations of the two buckwheat inflorescence colors revealed that adult *F. intonsa* showed a significant selection for the *F. tataricum* flower’s color (RGB: 254, 255, 245), while 2nd instar nymphs exhibited a highly significant selection. These findings underscored the critical influence of host plant floral coloration on host location and selection by *F. intonsa*.

On the other hand, the insect olfactory system plays a pivotal role in host plant selection. Insects possess a repertoire of olfactory-related proteins that bind to odorant molecules and facilitate their transport to corresponding receptors, thereby activating olfactory signaling pathways and enabling chemical communication [36,37]. Insects rely on their olfactory systems to locate and discriminate among host plants. Plant-derived volatile organic compounds (VOCs) not only influence insect selection, but also guide oriented movement and host recognition [38,39]. These VOCs consist of low-molecular-weight organic molecules released from leaves, fruits, flowers, and other plant parts during growth [40,41]. These volatile compounds, which include terpenoids, aldehydes, ketones, alcohols, and esters, are released by plants at specific stages throughout their growth periods [42,43,44]. These volatiles directly affect the behaviors of herbivorous insects, including feeding, mating, and oviposition, thereby significantly influencing insect development and fitness [45]. For instance, the strong selective response of *Plutella xylostella* (Linnaeus, 1758) (Diptera: Plutellidae) to cruciferous plants is closely associated with characteristic isothiocyanate volatiles [46]. Additionally, the (+)-α-Pinene was attractive to virgin males and females of *Gonipterus platensis* (Marelli, 1926) (Coleoptera: Curculionidae) at the lowest concentration tested from *Eucalyptus globulus* (Labill, 1800) (Myrtales: Myrtaceae) [47]. The present study employed orthogonal partial least squares-discriminant analysis (OPLS-DA) to profile volatile organic compounds from the inflorescences of white- and red-flowered buckwheat cultivars, identifying seven key volatiles. Among these, Δ-Cadinene exhibited pronounced attraction to 2nd instar nymphs and adults of *F. intonsa* across the tested concentration range. Furthermore, the response to Verbenone displayed concentration-dependent effects, with increasing concentrations resulting in progressively higher selection rates by both 2nd instar nymphs and adult thrips. Li et al. demonstrated that increased concentrations of Geraniol and 1-Octen-3-ol enhanced the attraction of *Thrips tabaci* (Linnaeus, 1889) (Thysanoptera: Thripidae) [48]. In their study, Isoamyl alcohol at a concentration of 10 μg/μL significantly attracted adult *F. intonsa*, while no significant differences were observed at other concentrations. Additionally, (S)-2-Methyl-1-butanol at 10 μg/μL exhibited remarkable attraction to 2nd instar nymphs and adult thrips, although this effect was not significant at lower concentrations. These studies indicate that the Δ-Cadinene and Verbenone released by buckwheat may attract *F. intonsa*. Previous reports have indicated that isoamyl alcohol attracted various lepidopteran insects, including *Mamestra brassicae* and *Amphipyra pyramidea* (Linnaeus, 1758) (Lepidoptera: Noctuidae) [49,50]. The conclusions drawn from this study align with those of earlier research. While this study focused solely on individual components of the volatile substances in buckwheat flowers, current investigations into the regulation of insect behavior by these compounds predominantly emphasize the combined analysis of volatile components [51,52,53].

Trap cropping, as an agroecological strategy for environmentally benign pest management, has been extensively validated in practical applications. Intercropping trap crops with main crops established a regional ecological pest management system [54,55], with common configurations including perimeter planting, inter-row intercropping, and relay cropping [56]. Related studies have demonstrated that the strategic deployment of trap plants around primary crops modified plant diversity and spatial configuration, which influenced pest survival, feeding behavior, and reproduction, ultimately curbing population expansion [57,58,59]. In this study, perimeter intercropping with two buckwheat varieties around sunflower plots significantly reduced field populations of *F. intonsa*. The white-flowered buckwheat exhibited markedly greater thrips trapping efficacy than the red-flowered buckwheat, which aligned with the choice assay results obtained under controlled conditions. During the reproductive stages of sunflower (R4-R7), the population of *F. intonsa* increased from August 6 to 19, indicating that the population growth rate of *F. intonsa* could have been increased exponentially during the blooming period (Figure 8). Yang et al. reported similar findings [60], where the population of thrips increased during the flowering period and peaked at full bloom. Li et al. [61] demonstrated that intercropping rosemary with sweet pepper significantly reduced the population densities of *F. intonsa*. Conversely, intercropping buckwheat and sunflower diminished *F. intonsa* populations in sunflower fields. It was plausible that buckwheat’s early flowering period attracted natural enemy insects of the *F. intonsa*. These natural enemies fed on *F. intonsa* present on the buckwheat, thereby expanding their populations. Subsequently, these natural enemies exerted a controlling effect on *F. intonsa* in the sunflower fields, leading to a reduction in population size. Further research on natural enemy insects is thus necessary.

In summary, this study clearly identified the significant attractiveness of white-flowered buckwheat to *F. intonsa* through cage selection, olfactory response, color tendency response, and volatile compound response. Furthermore, the population density of *F. intonsa* in sunflower-growing areas decreased, to some extent, when planting buckwheat in sunflower fields. These results provide a scientific foundation for the future promotion of white-flowered buckwheat as an attractive plant within the sunflower planting system.

## Figures and Tables

**Table 1:** Choice of *F. intonsa* between two color cards.

Test Insect	Instar	Selection of *F. tataricum*	Selection of *F. esculentum*
*F. intonsa*	2nd instar nymph	64.75% A	35.25% B
	Adult	59.79% a	40.21% b

Note: Different lowercase letters in the same row indicate significant differences (*p* < 0.05), while different uppercase letters indicate extremely significant differences (*p* < 0.01).

## Data Availability

The original contributions presented in this study are included in the article. Further inquiries can be directed to the corresponding author.
